# Physician-assisted suicide, euthanasia and palliative sedation: attitudes and knowledge of medical students

**DOI:** 10.3205/zma001010

**Published:** 2016-02-15

**Authors:** Johanna Anneser, Ralf J. Jox, Tamara Thurn, Gian Domenico Borasio

**Affiliations:** 1 TU München, Klinikum rechts der Isar, Klinik für Psychosomatische Medizin und Psychotherapie, Palliativmedizinischer Dienst, München, Deutschland; 2 LMU München, Institut für Ethik, Geschichte und Theorie der Medizin, München, Deutschland; 3 Universität Lausanne, Centre Hospitalier Universitaire Vaudois (CHUV), Service de Soins Palliatifs, Lausanne, Schweiz

**Keywords:** Medical students, Physician-assisted suicide, Euthanasia, Palliativ, Sedation, Legal aspects, Undergraduate Medical Education

## Abstract

**Objectives: **In November 2015, the German Federal Parliament voted on a new legal regulation regarding assisted suicide. It was decided to amend the German Criminal Code so that any “regular, repetitive offer” (even on a non-profit basis) of assistance in suicide would now be considered a punishable offense. On July 2, 2015, a date which happened to be accompanied by great media interest in that it was the day that the first draft of said law was presented to Parliament, we surveyed 4th year medical students at the Technical University Munich on “physician-assisted suicide,” “euthanasia” and “palliative sedation,” based on a fictitious case vignette study.

**Method:** The vignette study described two versions of a case in which a patient suffered from a nasopharyngeal carcinoma (physical suffering subjectively perceived as being unbearable vs. emotional suffering). The students were asked about the current legal norms for each respective course of action as well as their attitudes towards the ethical acceptability of these measures.

**Results: **Out of 301 students in total, 241 (80%) participated in the survey; 109 answered the version 1 questionnaire (physical suffering) and 132 answered the version 2 questionnaire (emotional suffering). The majority of students were able to assess the currently prevailing legal norms on palliative sedation (legal) and euthanasia (illegal) correctly (81.2% and 93.7%, respectively), while only a few students knew that physician-assisted suicide, at that point in time, did not constitute a criminal offense. In the case study that was presented, 83.3% of the participants considered palliative sedation and the simultaneous withholding of artificial nutrition and hydration as ethically acceptable, 51.2% considered physician-assisted suicide ethically legitimate, and 19.2% considered euthanasia ethically permissible. When comparing the results of versions 1 and 2, a significant difference could only be seen in the assessment of the legality of palliative sedation: it was considered legal more frequently in the physical suffering version (88.1% vs. 75.8%).

**Conclusion:**

- The majority of the students surveyed wrongly assumed that physician-assisted suicide is a punishable offense in Germany. However, a narrow majority considered physician-assisted suicide ethically acceptable in the case study presented.
- Compared to euthanasia, more than twice as many participants considered physician-assisted suicide acceptable.
- There was no significant difference between personal attitudes towards palliative sedation, physician-assisted suicide or euthanasia in light of physical or emotional suffering.
- Educational programs in this field should be expanded both qualitatively and quantitatively, especially considering the relevance of the subject matter, the deficits within the knowledge of legal norms and the now even higher complexity of the legal situation due to the new law from December 2015.

## Zusammenfassung

**Zielsetzung: **Im November 2015 wurde im deutschen Bundestag über eine gesetzliche Neuregelung der „Sterbehilfe“ abgestimmt. Dies betraf insbesondere die rechtliche Bewertung des ärztlich assistierten Suizids. Es wurde eine Änderung des Strafgesetzbuches beschlossen, das die „geschäftsmäßige Förderung der Selbsttötung“ nun unter Strafe stellt. Bereits am 2.7.2015, dem Tag der von größerem medialem Interesse begleiteten ersten Lesung entsprechender Gesetzesentwürfe im Bundestag, befragten wir Medizinstudierende des 7. und 8. Semesters der TU München zu den Themen „ärztlich assistierter Suizid (ÄAS)“, „Tötung auf Verlangen (TAV)“ und „palliative Sedierung (PS)“ auf der Basis einer fiktiven Fallvignette.

**Methodik: **Die Fallvignette schilderte den Fall eines an einem Nasopharynxkarzinom erkrankten Patienten in zwei Varianten (subjektiv unerträgliches physisches vs. seelisches Leiden). Gefragt wurde nach der für die jeweiligen Handlungsoptionen aktuell geltenden Rechtslage sowie nach der Einstellung der Studierenden zur ethischen Vertretbarkeit dieser Maßnahmen.

**Ergebnisse: **241 von 301 Studierenden (80%) beantworteten den Fragebogen, davon 109 in der Variante 1 (körperliches Leiden) und 132 in der Variante 2 (seelisches Leiden). Die geltende Rechtslage zur PS (erlaubt) und zur TAV (verboten) wurde von der Mehrzahl der Studierenden richtig eingeschätzt (81,2% bzw. 93,7%), während nur eine Minderheit der Studierenden (18,8%) wusste, dass der ÄAS zum damaligen Zeitpunkt keinen strafrechtlich relevanten Tatbestand darstellte. Für – im geschilderten Fall – ethisch vertretbar hielten 83,3 % der Teilnehmer die PS unter gleichzeitiger Beendigung der Ernährung und Flüssigkeitsgabe, 51,2% den ÄAS und 19,2% die TAV. Im Vergleich der Varianten 1 und 2 fand sich ein signifikanter Unterschied lediglich bei der Einschätzung der Rechtmäßigkeit der PS: Bei physischem Leid wurde diese häufiger für erlaubt erachtet (88,1% vs. 75,8%).

**Schlussfolgerung:**

- Die große Mehrheit der befragten Studierenden ging zu Unrecht von einer Strafbarkeit des ÄAS in Deutschland aus. Eine knappe Mehrheit hielt den ÄAS im geschilderten Fall für dennoch ethisch vertretbar.
- Im Vergleich zur TAV hielten mehr als doppelt so viele Befragte ÄAS für akzeptabel.
- Es konnte kein signifikanter Unterschied in der persönlichen Einstellung zu PS, ÄAS oder TAV bei Vorliegen von physischem oder seelischem Leiden gefunden werden.
- Das Lehrangebot in diesem Bereich sollte wegen der Relevanz der Thematik, den Defiziten in der Kenntnis rechtlicher Grundlagen und der nun noch höheren Komplexität der rechtlichen Situation durch die Gesetzesänderung vom November 2015 qualitativ und quantitativ ausgebaut werden.

## 1. Introduction

Physician-assisted suicide and euthanasia play a major role in current bioethical discussions. The primary question is how assisted suicide, especially physician-assisted suicide, can or should be evaluated both ethically and legally. The bill by the intergroup surrounding the members of parliament Michael Brand and Kerstin Giese was accepted in the parliamentary vote on November 6, 2015 and resulted in a new §217 in the German Criminal Code. Those who “intentionally promote the suicide of another person and, in a regular and repetitive manner, grant, provide or facilitate the opportunity to do so” are now subject to a “prison sentence of up to 3 years” or a fine. Those who “do not act in a regular and repetitive manner” and are relatives or related parties remain exempt from punishment. Other bills, which would have granted doctors substantive criteria providing legal security for the legitimacy of physician-assisted suicide in individual cases, were not accepted.

### 1.1. Representative surveys in Germany

Numerous population surveys conducted by opinion research institutes concurrently showed that over the past few years the majority of German citizens (on average 72%) are of the opinion that physician-assisted suicide and euthanasia should be legal [9]. Furthermore, the comparison of two opinion polls by the Allensbach Institute from 2010 and 2014 shows that popular consent to “active euthanasia” tends to be increasing [http://www.bundesaerztekammer.de/fileadmin/user_upload/downloads/Sterbehilfe1.pdf], [http://www.ifd-allensbach.de/uploads/tx_reportsndocs/KB_2014_02.pdf].

#### 1.2. Surveys among doctors

The acceptance of physician-assisted suicide and euthanasia among doctors has been investigated in numerous studies: in a representative survey among German physicians that was commissioned by the German Medical Association in 2009 showed that 30% support legal permission to conduct physician-assisted suicide and 17% support the legalization of euthanasia; 37% would provide suicide assistance under certain circumstances [http://www.bundesaerztekammer.de/fileadmin/user_upload/downloads/Sterbehilfe1.pdf]. In a smaller survey [17], 47% of doctors had a positive attitude towards physician-assisted suicide and 32% of participants shared the same attitude towards euthanasia. In another and more recent study [10], 40.2% of the doctors surveyed could see themselves providing suicide assistance “under certain circumstances.” In a current survey among members of the German Association for Hematology and Medical Oncology, 34% of participants indicated that they would take “assisting a patient with suicide” into consideration “under certain circumstances” [13]. A survey among a representative sample of Bavarian physicians revealed that doctors that have been practicing medicine longer tend to be more in favor of physician-assisted suicide and euthanasia than their colleagues who have less professional experience [16].

#### 1.3. Surveys among medical students

Medical students constitute a special group within the current discussion. On the one hand, the legal regulation that has been enacted (and what has not been enacted) will influence their medical actions in the near future. On the other hand, it may be the case that this new generation of physicians has a different attitude towards these questions. Among other things, this may be due to the fact that, unlike most working doctors, they were required to attend courses on palliative medicine as well as medical ethics during their undergraduate medical studies. One of the main objectives of these courses is to provide students with the opportunity to attain their own reflected attitude on these issues. This includes teaching the necessary knowledge as well as providing appropriate teaching methods – for example, discussions in small groups. Previous studies have shown that medical students have a strong interest in ethical questions, but in their opinion, the amount and types of courses available are not sufficient [2], [17].

#### 1.4. Goal of the study

**Figure 1:**
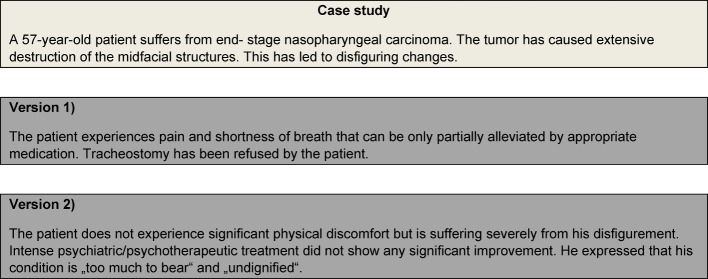
Case study in version 1 (physical suffering) and version 2 (emotional suffering)

Our goal was to investigate knowledge of the current legal situation as well as attitudes towards physician-assisted suicide, euthanasia and palliative sedation among a group of medical students. In order to prevent misunderstandings, which may occur due to the somewhat confusing terminology within the German language (so-called “passive,” “active,” “indirect,” “direct” euthanasia) [4], we decided to base our survey, which was conducted in German, on a case study (see figure 1 (Fig. 1)).

## 2. Methods

### 2.1. Participants

The participants were 4th year medical students at the Technical University Munich. After a written exam, the students were asked to complete a short questionnaire. In their previous year at medical school, all of the students were required to attend and complete courses on the ethics and history of medicine, on medical law as well as part of the palliative care curriculum.

#### 2.2. Questionnaire

The fictitious case vignette study, in which a patient suffers from a nasopharyngeal carcinoma, was presented in two versions (see figure 1 (Fig. 1)). The questionnaire with either version 1 or version 2 was randomly distributed among the students. According to version 1, the patient mainly suffers from untreatable physical symptoms (shortness of breath, pain). In version 2, the patient does not experience any significant physical discomfort, but an insufficiently treatable emotional, existential affliction that is a result of the illness. Each version of the case study subsequently presents three scenarios:

1. (palliative sedation) “Due to his/her physical or emotional suffering (after all possible treatment measures have been exhausted), the patient would like to receive palliative sedation. He/she does not want the administration of nutrition nor hydration during the sedation.”
2. (physician-assisted suicide) “The patient would like you to prescribe medication that he/she can take to end his/her life.”
3. (euthanasia) “The patient explicitly asks you to administer medication that will cause death.”

The students were asked about the (criminal) legality of each approach, as well as their personal attitudes. In addition, the participants’ age and gender were recorded. The study was approved by the Institutional Review Board of the Medical Faculty of the Technical University Munich (number 503/15).

#### 2.3. Data analysis

The data were evaluated using the statistics program IBM SPSS version 22. Group differences were determined using the χ^2^–Test and correlations were generated according to Spearman.

## 3. Results

### 3.1. Questionnaire response rate

Out of 301 students in total, 241 (80%) completed the questionnaire (68.5% female). On average, the students were 24 years old (SD±2.8). Version 1 of the questionnaire (physical suffering) was completed by 109 students and 132 students completed the version 2 questionnaire (emotional suffering). 

#### 3.2. Knowledge about legal norms

The majority of students (81.2%) correctly assessed the palliative sedation scenario as a legal treatment option, while only 18.8% rightly indicated that the physician-assisted suicide scenario was legally permissible. 93.7% of students rightly judged the euthanasia scenario as being a punishable offense (see table 1 (Tab. 1)).

#### 3.3. Attitudes towards the ethical acceptability of the treatment options

A vast majority of students (83.8%) opined that palliative sedation and the simultaneous withholding of nutrition and hydration was ethically acceptable in the given case study. A narrow majority (51.2%) was in favor of physician-assisted suicide, while one fourth of the students remained unsure (25.4%). Only 19.2% of the participants considered euthanasia an ethically acceptable treatment option, while more than half (56.9%) dismissed this option (see table 2 (Tab. 2)).

#### 3.4. Correlation between the acceptance of physician-assisted suicide and the acceptance of euthanasia

Students who considered physician-assisted suicide to be justifiable had varying opinions on euthanasia. The majority of students who supported physician-assisted suicide were against euthanasia (51 students), 43 students considered both procedures justifiable and 26 supporters of physician-assisted suicide were unsure about their opinion regarding euthanasia.

#### 3.5. Correlation between assumed permissibility of palliative sedation, physician assisted suicide and euthanasia with personal attitudes

The assumed permissibility and personal attitudes regarding the treatment options for palliative sedation and euthanasia were positively correlated (r=0.27 or 0.28; p<0.01). There was no such correlation for physician-assisted suicide.

#### 3.6. Physical vs. emotional suffering

When comparing version 1 and 2 (physical vs. emotional suffering), a significant difference could only be seen in the evaluation of the legality of palliative sedation: 88.1% of students considered palliative sedation permissible in the case of physical suffering, whereas only 75.8% considered palliative sedation legal in the case of emotional suffering (p=0.0139) (see table 1 (Tab. 1)).

There was no significant difference between versions 1 and 2 regarding the ethical acceptability of the three treatment options (palliative sedation, physician-assisted suicide, euthanasia) (see table 2 (Tab. 2)). The administration of palliative sedation was only slightly more frequently supported in the case of emotional suffering than in the case of physical suffering (88% vs. 80.3%). 

#### 3.7. Demographic differences

There was no significant difference between the answers given by female and male participants. In addition, there was no correlation between the answers provided and the age of the participants.

## 4. Discussion

### 4.1. Knowledge of legal norms

The results show that most of the medical students who took part in the survey were able to correctly assess the legal permissibility of palliative sedation and the status of euthanasia as a punishable offense, whereas a large majority did not know that physician-assisted suicide, at that point in time, was legal in Germany without any restrictions. In an older publication [17], it was reported that a high percentage of medical students erroneously considered permissible options of treatment limitation to be a criminal offenses. Thus, 62% wrongly assumed that withdrawing parenteral nutrition and hydration from dying patients was illegal. In contrast, a large majority of the students in our investigation correctly regarded palliative sedation in the case of a patient suffering from terminal cancer (with all disease-modifying treatment options exhausted) to be legal. The categorization of euthanasia as a criminal offense likewise did not cause any problems.

Students’ knowledge of the legal norms of physician-assisted suicide in Germany was investigated in several earlier studies: Clemens et al. [6] discovered that only one fifth of medical students were able to rightly indicate that physician-assisted suicide did not present any relevant criminal elements. Similarly, a smaller study asserted that 85% of medical students wrongly considered physician-assisted suicide to be an illegal measure [11].

The majority of students in our study still were not familiar with the legal classification of physician-assisted suicide, despite the popular debate on assisted suicide that has gained widespread media attention over the past few years. On the one hand, this may be due to the fact that, despite the unrestricted criminal permissibility in force at the time, the legal situation was difficult to understand, which was a result of various court rulings, the controversial discussion on guarantor duty and the restrictive narcotics law. In addition, the professional code of ethics for physicians may have had a major influence: the prohibition of physician-assisted suicide stated in the model code by the Federal Medical Association, as well as the corresponding prohibition in the legally binding codes of 10 out of 17 State Medical Associations, tend to reinforce the assumption that physician-assisted suicide is a fundamentally illegal action [http://www.gesetze-im-internet.de/_appro_2002/BJNR240500002.html].

The new legal regulation, effective as of December 2015, further complicates the situation: the new law stipulates that any “regular, repetitive offer” (even on a non-profit basis) of assistance in suicide will be punishable. It remains unclear whether physicians are liable for prosecution if they assist a patient in committing suicide, as any professional activity is by definition repetitive. Prior to the passing of the law, the scientific board of the German Federal Parliament criticized that the proposed bill did not sufficiently clarify how to differentiate between the regular, repetitive form of assisted suicide that becomes illegal and a form of assisted suicide that would be exempt from prosecution.

With regard to our study, it needs to be mentioned that the “practical training day for palliative medicine,” in which this particular topic is also addressed, takes place in the 5^th^ year and had thus not been experienced yet by the participants of this survey.

In accord with the results from the afore-mentioned studies, our data show that it still cannot automatically be assumed that medical students have the knowledge concerning relevant legal norms, especially with regards to physician-assisted suicide. However, this knowledge is a necessary prerequisite so that students can establish their own attitude and opinion towards these issues. Otherwise, there is the risk that end-of-life decisions, even among physicians in high-ranking positions, are made on the basis of insufficient or even false knowledge concerning the respective ethical and legal conditions [5]. 

#### 4.2. Physical vs. emotional suffering

The case study was presented to the students in two versions: physical vs. emotional-existential suffering. In general, it has already been shown that various factors influence the decision on euthanasia in fictitious case vignette studies [15]. Emotional-existential suffering has been controversially discussed, especially within the context of palliative sedation. On the one hand, a study showed that 13-29% of all palliative sedations are performed primarily on the grounds of psycho-existential suffering [3]. On the other hand, for many clinicians mental suffering “in itself” does not constitute an acceptable reason for palliative sedation [7]. According to the guidelines of the *European Association for Palliative Care* (EAPC), palliative sedation can also be “considered for severe non-physical symptoms such as refractory depression, anxiety, demoralization or existential distress”. However, it needs to be noted that there is “no consensus on the appropriateness of sedation for these indications” [1]. In our study there was no significant difference between the students’ ethical evaluation of physical and psycho-existential suffering. However, there was a difference in the evaluation of their legality: more students considered palliative sedation legally permissible if the patient exhibited inadequately treatable physical symptoms.

#### 4.3. Students’ personal attitudes

Even though the majority of students (72.6%) assessed physician-assisted suicide as being punishable, a narrow majority (51.2%) considered physician-assisted suicide ethically justifiable in the given case study. Thus, in comparison to similar surveys, medical students show a more favorable attitude towards physician-assisted suicide than doctors [10], [13], [17]. The combination of considering this treatment option both ethically acceptable as well as legally punishable could be seen among 34.4% of the participants [14]. 

In our study, only a minority of students was in favor of euthanasia (19.2%). This complies with the results of a study from 2004: only 20% of medical students surveyed in Berlin could imagine a situation in which they would be prepared to kill a patient [12]. Similar results could be seen in a survey by the German Medical Association from 2009; whereas in a study by Zenz et al. [18], 32.1% of the doctors surveyed expressed a favorable attitude towards euthanasia. The explicit rejection of euthanasia, as compared to physician-assisted suicide, signals that students – unlike the general population – detect a significant ethical difference between these two treatment approaches, a trend that has also emerged in more recent scientific literature [8]. Thus, the majority of students who were in favor of physician-assisted suicide were also of the opinion that euthanasia is ethically unjustifiable.

#### 4.4. Limitations of the study

One of the primary limitations of this study is that the survey was conducted at only one university and among students of one academic generation. Thus, the study is only a limited representation of German medical students. It is to be assumed that the student’s local characteristics and the practical teaching methods also influenced the results. The questionnaire was kept very short and further demographic data, such as religious affiliation or medical activities outside of academic studies, was not recorded in order to ensure the anonymity on this sensitive issue. One of the strengths of the study is the very high response rate of 80%.

## 5. Conclusion

The strong interest of medical students seen in teaching courses on this subject is reflected in the high response rate of this survey. Furthermore, only 0.8-2% of the students in our survey indicated that they had no opinion on the ethical justifiability of palliative sedation, physician-assisted suicide and euthanasia. However, their knowledge – especially regarding the legal conditions surrounding end-of-life decisions – needs to improve. A qualitative and quantitative augmentation of educational programs seems urgently necessary, particularly in view of the new law in Germany on this issue.

In light of this, we suggest:

1. introducing additional programs or the integration of existing educational activities to improve students’ knowledge of the current legal situation – preferably in a cooperation between teachers of medical law, ethics and palliative medicine;
2. improving communication skills through video-based practice conversations with standardized patients in order to enable the students to learn how to adequately communicate with patients and families about physician-assisted suicide, euthanasia or palliative sedation;
3. linking reflections on ethics to concrete practical experiences (e.g. in seminars on case-based reflections during their clinical year) in order to provide students with the opportunity to develop their own ethically reflected attitudes.

## Competing interests

The authors declare that they have no competing interests.

## Figures and Tables

**Table 1:**
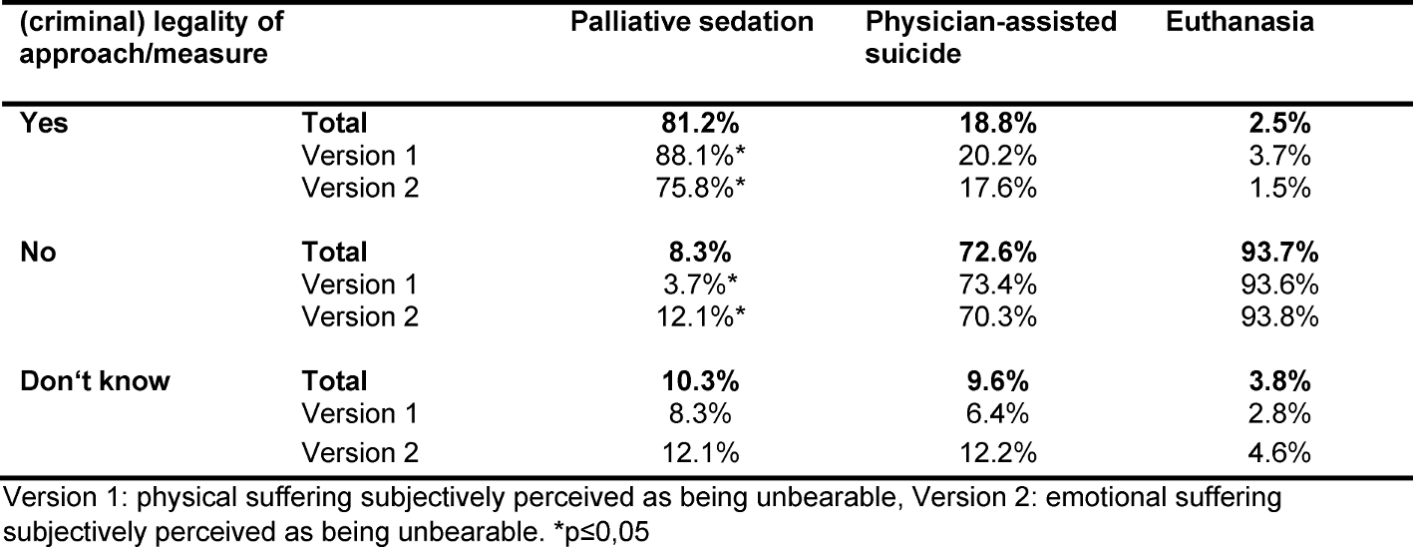
Currently prevailing legal norms on forms of euthanasia/assisted dying in Germany as assessed by medical students

**Table 2:**
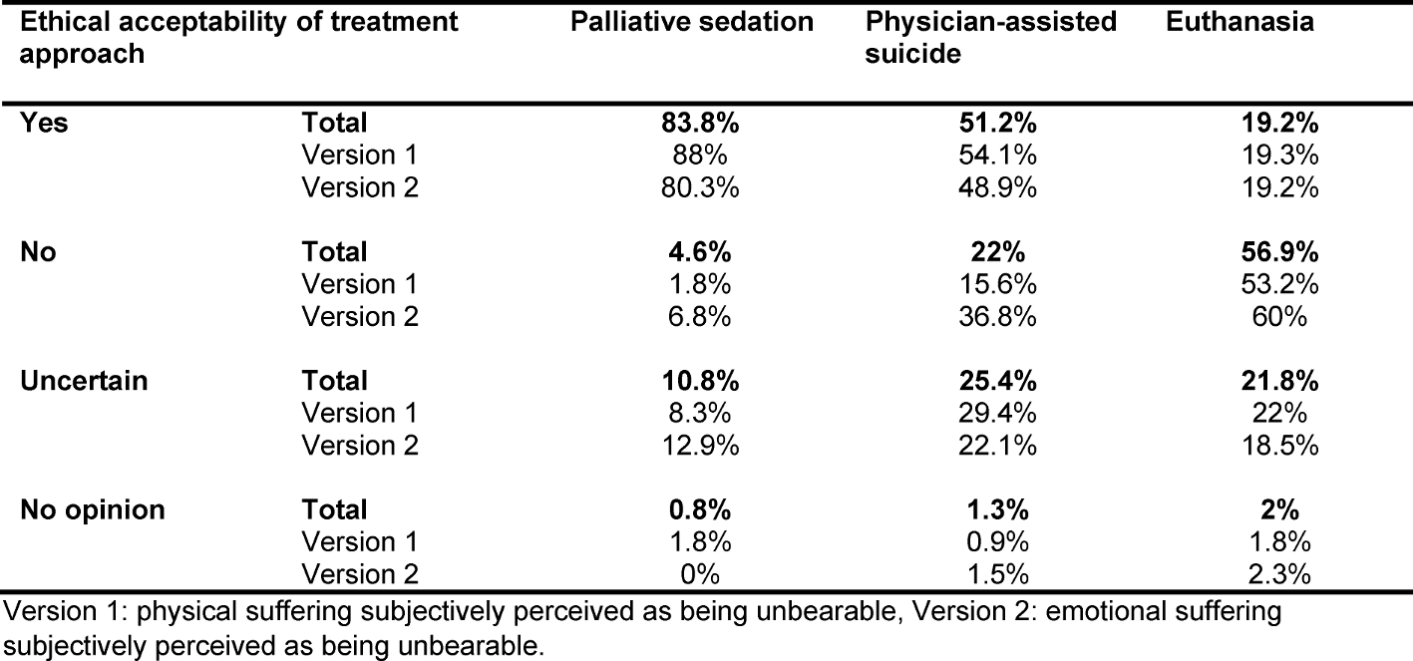
Ethical acceptability of options “palliative sedation”, “physician-assisted suicide” and “euthanasia” as assessed by medical students
